# User Based Development and Test of the EXOTIC Exoskeleton: Empowering Individuals with Tetraplegia Using a Compact, Versatile, 5-DoF Upper Limb Exoskeleton Controlled through Intelligent Semi-Automated Shared Tongue Control

**DOI:** 10.3390/s22186919

**Published:** 2022-09-13

**Authors:** Mikkel Berg Thøgersen, Mostafa Mohammadi, Muhammad Ahsan Gull, Stefan Hein Bengtson, Frederik Victor Kobbelgaard, Bo Bentsen, Benjamin Yamin Ali Khan, Kåre Eg Severinsen, Shaoping Bai, Thomas Bak, Thomas Baltzer Moeslund, Anne Marie Kanstrup, Lotte N. S. Andreasen Struijk

**Affiliations:** 1 Center for Rehabilitation Robotics, Department of Health Science and Technology, Aalborg University, 9220 Aalborg, Denmark; 2 Department of Materials and Production Technology, Aalborg University, 9220 Aalborg, Denmark; 3 Visual Analysis and Perception (VAP) Lab, Department of Architecture, Design, and Media Technology, Aalborg University, 9000 Aalborg, Denmark; 4 Department of Planning, Aalborg University, 9000 Aalborg, Denmark; 5 Spinal Cord Injury Centre of Western Denmark, Viborg Regional Hospital, 8800 Viborg, Denmark; 6 Department of Electronic Systems, Aalborg University, 9220 Aalborg, Denmark

**Keywords:** assistive technology, exoskeletons, human-robot interaction

## Abstract

This paper presents the EXOTIC- a novel assistive upper limb exoskeleton for individuals with complete functional tetraplegia that provides an unprecedented level of versatility and control. The current literature on exoskeletons mainly focuses on the basic technical aspects of exoskeleton design and control while the context in which these exoskeletons should function is less or not prioritized even though it poses important technical requirements. We considered all sources of design requirements, from the basic technical functions to the real-world practical application. The EXOTIC features: (1) a compact, safe, wheelchair-mountable, easy to don and doff exoskeleton capable of facilitating multiple highly desired activities of daily living for individuals with tetraplegia; (2) a semi-automated computer vision guidance system that can be enabled by the user when relevant; (3) a tongue control interface allowing for full, volitional, and continuous control over all possible motions of the exoskeleton. The EXOTIC was tested on ten able-bodied individuals and three users with tetraplegia caused by spinal cord injury. During the tests the EXOTIC succeeded in fully assisting tasks such as drinking and picking up snacks, even for users with complete functional tetraplegia and the need for a ventilator. The users confirmed the usability of the EXOTIC.

## 1. Introduction

Each year, between 250,000 and 500,000 individuals worldwide are believed to suffer a spinal cord injury (SCI) [1]. In Northern America alone, SCI cases account for approximately 250,000 individuals [2], of which more than half suffer from tetraplegia [3] with all four limbs being affected. When a high-level complete SCI occurs, the injured individual may not be able to move from the neck down (complete functional tetraplegia). This devastating condition can lead to a loss in quality of life [4] as well as a reduced life expectancy [1]. Further, approximately 22% of individuals with SCI suffer from depression and premature death [5].

Furthermore, individuals with tetraplegia often require constant care from health professionals and a single individual with complete functional tetraplegia may need a team of up to eight caregivers. Meanwhile, human resources from the health care sector are increasingly sparse as the aging world’s demography is bound to require more resources within the next decades [6]. This poses an urgent need for solutions that empower individuals with severe disabilities while potentially freeing resources in the health care sector.

An approach to achieve this goal is to apply robotic assistive technologies. A study by Maheu et al. [7] found that introducing an assistive robotic manipulator (ARM) for individuals with upper limb disabilities could reduce the need for help by up to 41%. Consequently, upper arm exoskeletons hold great potential as assistive devices for individuals with severe paresis or paralysis as a tool to regain some independence [8].

**Figure 1:**
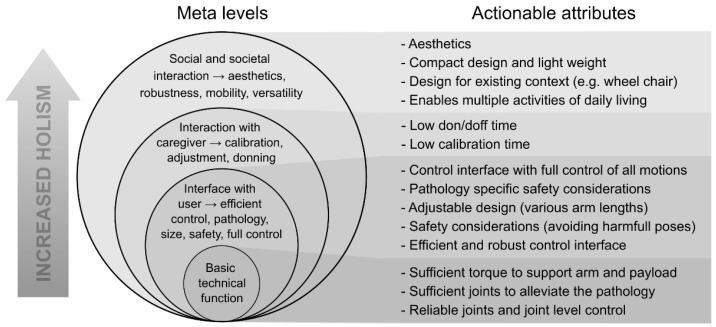
Onion model representing the meta-levels of design considerations for assistive exoskeleton design. To the right, attributes of each level are specified into actionable attributes. Note: the figure should be read bottom up.

Recently, significant advances have been seen in upper limb exoskeleton (ULE) design [9]. However, generally applicable systems with a critical level of usability that enables domestic assistance of multiple activities of daily living (ADL) for individuals with complete functional tetraplegia are still lacking. To be able to support a user with complete or severe functional tetraplegia in performing multiple ADLs in domestic settings, several critical attributes of the exoskeleton system should be considered. Interdisciplinary development of ULEs including user involvement, involvement of clinicians, biomedical engineers, mechanical engineers, and electrical engineers may help reveal the multifaceted nature of these attributes. Based on our experience from such an interdisciplinary study, we have identified several critical attributes for domestic use and the potential adoption of ULEs in the everyday life of individuals with complete functional tetraplegia. These attributes are summarized in the onion model shown in Figure 1. For an assistive ULE to be fully useful as a domestic assistive device, it should support a large variety of motions and ADLs in a manner where the user is always in control and without compromising the social identity, the safety, or the health of the user. Further, the system should be fast to mount and calibrate and it should be mobile.

The existing literature on upper arm exoskeletons focuses mostly on exoskeletons for industrial or rehabilitation purposes [10]. Rehabilitation exoskeletons are often advanced high degree of freedom (DoF) exoskeletons that could provide good support in assistive applications, but they mostly suffer from two major obstacles: “bulkiness” and lack of mobility [9,11,12,13,14]. However, there are exceptions such as the Recupera [15,16], which is mobile and compact but protrudes significantly from the wearer’s arm. Often, industrial exoskeletons only actuate a subset of DoFs needed to perform ADLs, which renders them incapable of supporting individuals with complete tetraplegia.

A few systems have been developed for physical assistance, especially for individuals with weakened physiques [9,17,18,19]. However, only three systems have been found which focus on providing assistance and which can potentially support ADL tasks for individuals with tetraplegia: The HAL-UL [20], the “mobile wearable upper-limb exoskeleton” [21], and the NESM exoskeleton [22,23]. However, the HAL-UL lacks a wrist supination/pronation joint, which means that it can only grab objects that are standing upright, thereby limiting the number of tasks it can perform. Contrary to the HAL-UL, the mobile wearable upper-limb exoskeleton has four DoFs and is compact, but the order and type of joints in the kinematic chain limit the range of motion due to singularities such that if the elbow is flexed, it is impossible to pronate/supinate the wrist and a similar problem is true for the upper arm internal/external rotation. Additionally, both exoskeletons have been created with power assistance in mind rather than complete arm support and they require active movement of the arm or muscle activity to operate them, which individuals with complete functional tetraplegia are not capable of.

The NESM exoskeleton [22,23] has been designed for individuals with stroke and features five DoFs in the arm and four DoFs in the hand and wrist enabling a good range of motion and full arm assistance. While it can likely perform many ADLs for individuals with tetraplegia, it has a considerable size and weight. Although Crea and Nann et al. [22,23] showed good results testing it on individuals with stroke, it used a combined encephalography (EEG) and electro-oculography (EOG) brain–machine interface (BMI) that, despite its efficient recognition of commands, essentially acted as a start signal to a preprogrammed movement. In addition to being used within rehabilitation, this system has great potential in assistance of users with, e.g., late-stage amyotrophic lateral sclerosis (ALS) or locked-in syndrome, in which the interface options are limited. A similar exoskeleton has been presented by Barsotti et al. [24], but it suffers from the same shortcomings in relation to the control interface and is not wheelchair mountable. Table 1 summarizes some of the attributes of these exoskeleton systems.

While exoskeletons hold great promise for individuals with severe disabilities, a paradox arises as the disabilities at the same time make it harder for individuals to operate such assistive technologies. For example, an individual with paralysis or severe paresis in the arms is not able to use, e.g., arm movements, a joystick, or pushbuttons as control input for an exoskeleton.

Currently, existing interfaces for individuals with complete functional tetraplegia consist of either chin sticks [25], sip and puff, voice activation, eye-tracking [26], BMI [22,23,27,28] or combinations thereof [29]. Common for these interfaces is that they exhibit one or more of the following: being indiscreet; being inefficient in terms of the time needed to activate a command; being inflexible in the sense that they only allow for a very limited number of command inputs, or they require substantial and or repeated calibration.

To increase the efficiency of assistive devices, autonomous functionality is often applied [30,31], which can perform some portion of the motions for a given task. Indeed, this can increase performance and accuracy [31,32]. Kim et al. [31] found that the way in which these functions are invoked is likely of high importance, especially for individuals with physical disabilities. Individuals with SCI found it less satisfying to initiate autonomous execution of a task with an ARM, as opposed to completing the given task manually, due to the reduced sense of agency. This is despite a worse performance using manual control. Hence, autonomous functionality can increase performance, but the way in which it is implemented is of great importance, as users prefer to be in control at all times [31].

Finally, minimal attention has been given to uncovering the opinions, desires, and concerns of potential end-users of the exoskeletons [33,34]. Therefore, we have previously developed methods to engage users in the design of the exoskeleton arm and conducted interviews and workshops with nine adults living with severe tetraplegia [35,36]. Complemented by user experiences from experiments with a tongue-controlled assistive robot [37], these interviews showed that the users prioritized being able to drink and eat on their own. Not necessarily in the sense of consuming a whole meal but rather during repetitive activities such as eating fruit or candy (snacking) while, e.g., watching television; this to avoid constantly having to ask for assistance to obtain yet another piece. Another important insight was that the time necessary to don and doff the exoskeleton arm was of high priority as the addition of further time might down-prioritize mounting the exoskeleton and potentially lead to abandonment [34,38]. Regarding size and function, the interviewees prioritized function but emphasized that a balance between function and size was paramount [36,38].

Apart from user desires, special attention must be paid to tetraplegia as a medical condition. In particular, individuals with SCI-related tetraplegia are prone to exhibit autonomic dysreflexia (AD); a sudden attack in which the blood pressure rises dramatically, and which can be fatal in rare circumstances [39]. AD can be triggered by pressure applied to the skin or excessive skin stretching. In addition, individuals with SCI may suffer from spasticity. These issues may add requirements to safety and the attachment between the exoskeleton and the user.

**Figure 2:**
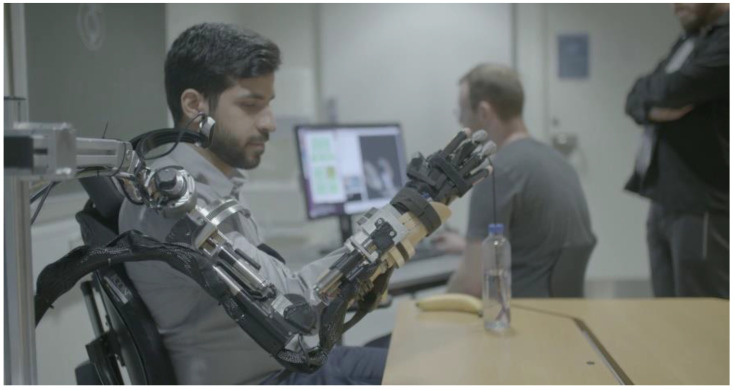
The EXOTIC exoskeleton system. An individual commands the EXOTIC exoskeleton using a tongue interface shared with computer vision-based semi-automation. The individual shown in the image has given written consent to the use of the image.

In this paper, we present the EXOTIC exoskeleton system, see Figure 2, in which we have strived to include all the considerations and challenges listed in each layer of the model presented in Figure 1. To evaluate the proposed system, it was tested with ten able-bodied individuals and three individuals with severe to complete functional tetraplegia. The tests comprised four ADL tasks inspired by a qualitative investigation of the desires and needs of individuals with severe tetraplegia [38]. The EXOTIC exoskeleton system is the first exoskeleton system empowering users with complete functional tetraplegia to perform arbitrary motions and multiple prioritized ADLs independently and efficiently while continuously being in control of the exoskeleton through intelligent shared tongue control. Table 1 compares existing ULEs with the proposed EXOTIC exoskeleton system.

**Table 1:** An overview of existing exoskeletons and which attributes they fulfill/include. Green shading indicates that the given attribute was fulfilled/included. Selection criteria: (1) must fully support a paralyzed arm, (2) must implement arm (min. shoulder and elbow) actuation and hand (grab) actuation, (3) must be tangible/tested (i.e., no simulations), (4) must be wheelchair mountable (within reason). Abbreviations: VI = based on visual inspection of images, NA = not announced, NR = not relevant, CFT = complete functional tetraplegia, ADL = activities of daily living. * Powered wheelchairs use 24 V power sources.

	Attributes:	Enables Multiple ADLs in Individuals w. CFT	Efficient, Robust, and Continuous Control Interface Useable by Individuals w. CFT	Individuals w. CFT Can Control All Motions Fully	Calibration Time	Designed for Existing Context *	Computer Vision Based Semi-Automation	Compact and Light Exoskeleton Design	Aesthetical Concerns (Social Context)	CFT Pathology Specific Safety (AD)	Safety Considerations (Safe Human Operation)	Tests Performed	Adjust-Able Design	Basic Technical Functions
Studies:														
The EXOTIC exoskeleton system for tetraplegia (This study)		Multiple ADLs demonstrated in individuals w. CFT	Intraoral, efficient (0.76 s to start command [36]), robust interface (not affected by environment and commercially available).	Full manual control over all DoFs	Short tongue interface calibration (<1 min)	Wheelchair mountable, wheelchair battery-powered (24 V).	Simultaneously available shared control w. semi-automation based on computer vision and manual control	Compact, Motors parallel to arm, inner rotational joints encircle arm (3.7 kg)	“Invisible” interface, Compact exoskeleton design	Two strap wrist/palm. Open Orthopaedic braces. Loose mounting to avoid AD.	Physical stoppers to limit joints, wheelchair battery operated, current limited, open-brace design (easy to pull out), only moves while being commanded.	Four ADLs on ten able-bodied and three individuals w. CFT.	Adjust-able link lengths	4 arm DoFs, 1 hand DoF
EEG/EOG semi-autonomous exoskeleton for stroke (Nann et al. [22], Crea et al. [23])		One ADL demonstrated in individual w. chronic stroke. (drinking task)	EEG/EOG-based interface (1.43 s to initialize command). EEG is sensitive to multiple factors (EMI, biological)	Semi-automated state-based control only	Longer than EXOTIC (6 min and 18 s calibration time)	Wheelchair mounted. Power requirement NA.	Only operates with semi-automation based on computer vision.	Larger than the EXOTIC system (13 kg)	VI: Visible, protruding interface, larger exoskeleton.	VI: Two strap, arm/hand, open brace design.	Physical stoppers to limit joints, open-brace design (easy to pull out), veto signal (users able to send a stop signal), SEA joints.	One ADL on seven able-bodied and five individuals w. chronic stroke. None w. CFT.	Adjust-able link lengths	5 arm DoFs, 4 hand DoFs
Recupera exoskeleton for stroke rehabilitation (Kirchner et al. [15] Kumar et al. [16]).		No ADLs demonstrated (only exercises).	NR/Relies on residual movement	NR/Relies on residual movement	NR/No relevant user interface to assess.	Wheelchair mounted. Custom 48 V batteries for power	NA/NR	VI: Compact, but protruding significantly from lower arm (4.3 kg)	VI: Compact but protruding. No relevant user interface to assess.	VI: Two strap, upper/lower arm, open brace design.	Physical stoppers, battery operated, current limited. No relevant user interface to assess.	Exercises w. one able-bodied individual one w. chronic stroke. None w. CFT.	Adjust-able arm links	5 arm DoFs, 1 hand DoF
HAL-UL exoskeleton for assisting the elderly (Otsuka et al. [20])		One ADL demonstrated in able-bodied individual. (drinking task)	NR/Relies on residual movement	NR/Relies on residual movement	NR/No relevant user interface to assess.	VI: Likely wheelchair mountable. Power requirement NA.	NA/NR	VI: Compact, Motors parallel to arm, inner rotational joints encircle arm (weight NA)	VI: Compact exoskeleton design. No relevant user interface to assess.	VI: Wrist strap. One open, one closed brace	Physical stoppers. No relevant user interface to assess.	One ADL on an able-bodied individual. None w. CFT.	NA	4 arm DoFs, 1 hand DoF

## 2. System Design

### 2.1. Overview

The EXOTIC exoskeleton system consisted of three main elements: (1) the exoskeleton; (2) a tongue control interface (TCI); and finally, (3) a computer vision guiding system.

### 2.2. Exoskeleton Design

The core design goals for this exoskeleton were to fully assist upper arm motions enabling simple ADLs for users with tetraplegia and at the same time reduce the “bulkiness” and accommodate the user’s desires. This included relatively easy donning and doffing and mitigating the risk of provoking AD through ergonomic mounting.

To remedy the problem of “bulkiness”, various strategies can be employed such as: using Bowden-tube cable drives to move the actuation mechanisms towards the base frame or completely off the exoskeleton [40,41]; using flexible materials with discreet pneumatic actuators or cable drives [42], or simply by reducing the DoFs [17]. Each of these approaches has its advantages but comes with limitations. Flexible exoskeletons can be compact, to the point where they can be worn underneath clothes [43], and thus could be an ideal solution to the problem of “bulkiness”. However, they suffer from non-linear actuation, and challenges in getting positional feedback from the joints make reliable closed-loop control difficult. A reduction of the DoFs has obvious implications for the flexibility of the exoskeleton but provides a simple way to reduce the “bulkiness” at the cost of function. The human arm has seven main DoFs, which would ideally be needed in an exoskeleton arm to achieve a workspace similar to that of the human arm. However, to reduce the “bulkiness” of our exoskeleton, we chose to focus on the gross motions of the arm that are necessary for simple ADLs. These motions can be performed mainly through the shoulder extension/flexion, upper arm internal/external rotation, elbow flexion/extension, and finally wrist supination/pronation. Whereas the omission of the shoulder abduction/adduction is limiting the flexibility, a previous biomechanical study conducted at our lab [44] investigated the effects of locking the shoulder abduction/adduction joint and showed that the joint was not necessary to perform simple ADLs. In addition to the obvious advantage of reducing the “bulkiness” around the shoulder gained from omitting the shoulder adduction/abduction, this also has other advantages. For instance, it ensures that the arm of the user stays within the fixed boundaries of the wheelchair, thus avoiding potential harm to the immediate environment and the user. A passive abduction/adduction shoulder joint was added to afford a more natural pose. This joint was fixed to have an abduction angle of 20°.

**Figure 3:**
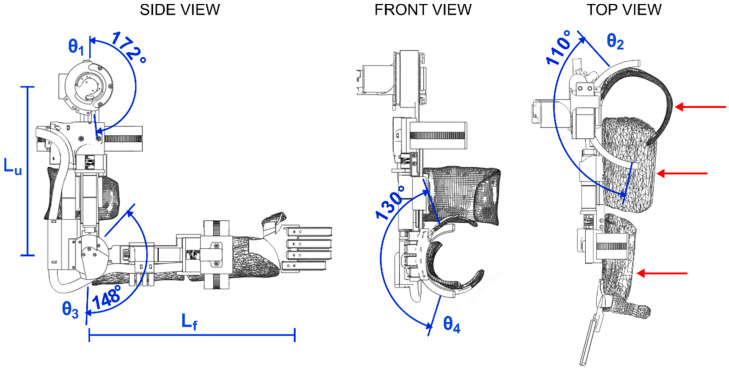
Detailed drawings of the EXOTIC exoskeleton and the range of motion of the four main degrees of freedom contributing to gross motion. From left: side view with shoulder (θ_1_) and elbow (θ_3_) flexion/extension and indications of link lengths (L_u_ and L_f_), which are adjustable; front view with wrist (θ_4_) supination/pronation; top view with upper arm internal/external rotation (θ_2_) and red arrows that indicate the three braces used to support the arm. Notation corresponds to the Denavit–Hartenberg parameters given in Table 2.

**Figure 4:**
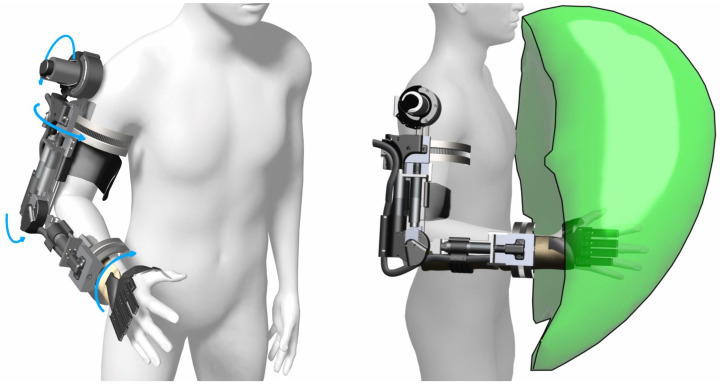
Three-dimensional renders of the exoskeleton with light blue arrows indicating the rotational joints and the available workspace indicated by the green volume on the right. Simulation was performed by permutating the joint angles over the range of motion of each joint and recording the hand location. The visualization was created from an approximation of the bounding volume of the resulting point cloud.

To mitigate the medical concern regarding AD, a set of three ergonomic braces were used, of which two were manufactured by an orthopedist (SAHVA A/S, Brøndby, Denmark). As opposed to strapping the arm in, the orthopedic braces “carried” the arm instead, see Figure 1, Figure 3, and Figure 4, akin to how an end-effector arm support supports the arm [45]. This provided a “loose” connection between the human arm and the exoskeleton, allowing the arm to adjust in the exoskeleton, thus reducing the risk of applying excessive pressure or twisting the skin of the user, which can cause AD. Additionally, this ensured a relatively fast donning and doffing during which the user’s arm was simply lifted into the three ergonomic braces. Two straps were used at the wrist and palm to secure the position of the hand relative to the exoskeleton.

The exoskeleton frame was created using a custom fabricated aluminum (7075) frame with variable link lengths to accommodate different arm lengths. To actuate the upper arm rotational joint, elbow flexion/extension joint, and wrist pronation/supination joints, three motors with planetary gears were fitted (EC-4pole, Maxon motor ag, CH), see Figure 3. Actuation of the shoulder flexion/extension was realized through a more powerful motor (EC-i40, Maxon motor ag, CH) and a strain wave gear (Harmonic Drive LLC, Beverly, MA, USA). The shoulder and elbow flexion/extension joints were actuated directly on the joint axis, whereas the rotational joints located on the axis of the arm were actuated through two dovetail, half-circular ring designs with the actuator actuating a gear that turned a semi-circular teeth-set [46], see Figure 2 and Figure 4. The joints were practically non-backdrivable except for the shoulder flexion/extension. However, this could only be backdriven under heavy load. The exoskeleton could be mounted onto wheelchairs and was designed to be powered by regular wheelchair batteries (24 V). Perspective 3D renders of the exoskeleton and a simulated workspace are shown in Figure 4. The Denavit–Hartenberg parameters of the four main DoFs of the exoskeleton are shown in Table 2 and visualized in Figure 3. While the focus of this paper is primarily on the overall user-based system design and particularly on the experimental side of our five DoF intelligently tongue-controlled exoskeleton systems as in [20,22], kinematics and modeling such as in [47,48], are available for a similar exoskeleton in Gull et al. [46].

Each motor was fitted with incremental encoders (>500 counts per rotation) for accurate motor control with sinusoidal commutation. Joint-level angular control was provided through miniature magnetic absolute encoders (RLS Merilna tehnika d.o.o., Sl) added directly to the shoulder and elbow flexion/extension joint axes. As the direct attachment of encoders to the joint axis of the internal/external shoulder rotation and the wrist pronation/supination was not possible due to the joint axis being internal to the arm of the wearer, a custom gearing was used on these encoders to extract the joint angles. Detailed drawings of the exoskeleton frame are shown in Figure 3.

The tendon-based soft-exoskeleton glove, CarbonHand (Carbonhand^®^, Bioservo Technologies AB, SE, Stockholm, Sweden), was used to provide one DoF for grasping objects. As the CarbonHand glove only enabled grasping and not actively released the grasp, a set of fabric elastic bands were used to counteract the grasping of the glove such that when the tendons were relaxed, the elastic bands would pull the hand back into an open pose.

### 2.3. Control Interface

Of the available interfaces for individuals with tetraplegia, chin sticks are reliable control inputs but are limited in the number of available commands and unattractive due to their indiscretion; if the users are even able to command them. Similarly, sip and puff systems are reliable but have a limited number of command inputs and are indiscrete. Conversely, eye-tracking can accommodate many simultaneous commands and can be useful when no other options are available, but it occupies the user’s gaze and attention and moreover the reliability can be a challenge [25]. Lastly, BMIs are particularly important for users who are paralyzed throughout the body as seen in the late stages of ALS. BMIs have recently shown promising results for exoskeleton control in laboratory settings [27]. However, BMIs often require substantial calibration, may be invasive [27], and are still too complicated to render applicable in everyday domestic settings for the continuous control of many DoFs. However, state-based control of exoskeletons using BMIs has recently shown promising results in assistive applications [22,28], even outside the laboratory [28].

**Figure 5:**
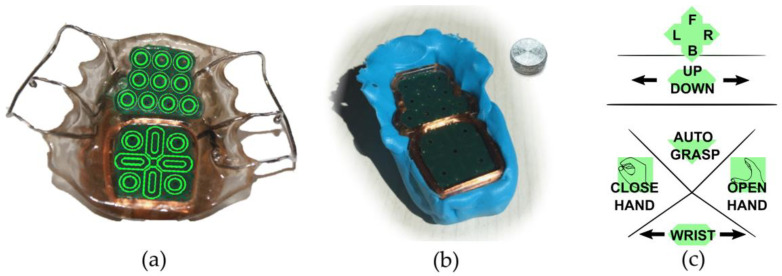
The iTongue tongue interface. (**a**) A standard commercial iTongue mouthpiece with sensor placements superimposed, (**b**) a temporary silicon palate brace as used in the current study together with an activation unit, and finally, (**c**) the control layout developed in this study to control the exoskeleton with shared computer vision based semi-automation. The topmost part acts as a joystick to control the position of the exoskeleton in a horizontal plane, while the up/down slider controls the vertical axis. The wrist supination/pronation is controlled through the bottommost slider. The remaining three icons on the interface act as simple push-and-hold buttons. Note: the text size in the layout has been increased in the figure to increase readability.

To address the problem of providing a reliable interface for individuals with tetraplegia, research into user interface technology is ongoing and has produced the intraoral tongue control interface, which was originally developed in our group [49,50], see Figure 5. As the name suggests, this interface allows the user to control technology through movements of the tongue. In particular, the tongue control interface has proven to be powerful, allowing individuals with tetraplegia to control a wide range of assistive solutions, including robots [51,52,53,54,55]. The intraoral tongue control system consists of an inductive sensing device embedded in a palate brace that works in conjunction with an activation unit attached to the tongue. The inductive sensing area detects the position of the activation unit, which corresponds to the position of the tongue. This allows the interface to have several simultaneously available command inputs. In effect, this can be used for direct control of several DoFs. The control takes place without the need for visual feedback in the form of, e.g., a screen as the user is able to feel where the tongue is, given that the user is trained in using the system.

**Figure 6:**
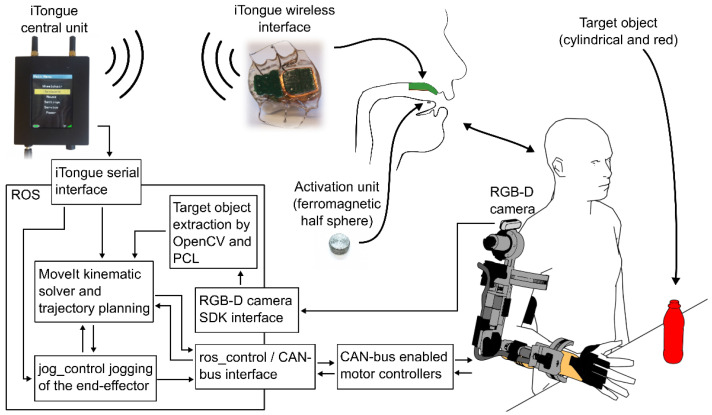
System overview. From the right bottom: The exoskeleton communicates via CAN-bus with a computer. From here all sensors of the exoskeleton are read and all motors are controlled. The RGB-D camera on the shoulder feeds its images through a computer vision pipeline resulting in a relative position and orientation of a target object (red bottle) enabling autonomous control. The user commands the exoskeleton through the adapted iTongue mouth unit. The wireless signals are received by the iTongue central unit, which in turn sends the received commands through a serial connection to a PC running the ROS ecosystem. Linear and square arrows indicate communication, double arrows indicate bidirectional communication. Note: the exoskeleton glove used in this study is not shown in this overview, but it is controlled through a USB serial interface.

The intraoral control interface used in this study was an adapted version of the commercially available CE-certified wireless iTongue device (iTongue^®^, TKS A/S, Nibe, Denmark) [42,43] as it enables an “invisible” and unintrusive way to control the exoskeleton, see Figure 6.

The mouthpiece of the iTongue is self-contained with a battery, onboard processing, and a wireless radio allowing it to be completely concealed in the mouth. It features 18 sensor areas that can be programmed to act as individual buttons or be interpolated to create larger virtual buttons and joysticks [37], see Figure 5 for a visual representation of the interface and its sensors and layout. The wireless signals are picked up using a central unit which conveys the signals to a computer for further processing.

### 2.4. Computer Vision-Based Shared Control System

Whereas the iTongue tongue control system is a versatile control method, it is a two-dimensional control interface meaning that a maximum of two degrees of freedom (DoF) can be controlled intuitively at the same time, for example for controlling an end-effector in a 2D plane. By adding intelligence to the control through intention prediction and spatial awareness, the same interface can be used to enable simultaneous control of more degrees of freedom through shared semi-automation. This approach has previously been applied for ARMs [30,31,32] and recently in an exoskeleton glove using different interfacing methods [22]. In effect, this allows a single button to control an exoskeleton with an arbitrary number of DoFs to guide it towards an object of interest.

In some previous systems [22,30,31], autonomous functionality was integrated with a “point and click” approach, e.g., pointing to an object of interest, clicking, and letting the robot/exoskeleton perform the desired action without any user opportunity to stop the system, potentially causing harm [56,57]. However, this approach has two caveats: the individual operating the device may feel that it is acting on its own accord and thus may feel distanced from it rather than identifying with the device [31]. In the case of the EXOTIC, this point is particularly important as the sense of empowerment gained from performing and achieving with the exoskeleton may in turn be paramount to avoiding abandonment of the technology [38]. Secondly, if the control algorithms command the exoskeleton to perform unintended movements or the movement is not entirely correct, the “point and click” method may mean that the device cannot be stopped before reaching its destination. The optimal strategy seems to be shared control, in which the user is always in control while autonomy assists in performing the movements [30,31].

To overcome these caveats, the intelligent control system presented here acted as a “push and hold” function, such that the user was to command the exoskeleton to move continuously until the user deemed the motion complete or wanted to stop the motion in case the exoskeleton was not moving as intended. If the latter was the case, the user could manually correct the motion using direct manual control. Not only did this ensure that the user was always in control, but it also increased safety considerably [56,57].

To enable spatial awareness and semi-autonomous control, a color and depth camera (Intel^®^ RealSense™ D415, Intel Corporation, Santa Clara, CA, USA) was added to the EXOTIC exoskeleton system. The camera was placed above the shoulder joint, see Figure 6. The camera pointed towards the workspace in front of the exoskeleton enabling the use of computer vision algorithms to locate the position and orientation of objects of interest. An overview of the system is shown in Figure 6.

## 3. Methods

### 3.1. Exoskeleton Control

The actuators located at each joint on the exoskeleton were controlled through CAN-bus-enabled motor controllers (EPOS4, Maxon motor ag, Sachseln, Switzerland, CH). Each motor controller was connected via a CAN-bus USB interface (USB-CAN-SI-M, TITAN Electronics Inc., Taoyuan City, Taiwan) allowing the exoskeleton to be controlled from a PC, see Figure 6.

The encoders on the joints were connected to the motor controller modules allowing the use of a built-in PID control (autotuned). Communications were handled through the CAN-bus network management (NMT) service setting the motor controller modules as NMT slaves, while a computer with the USB to the CAN-bus dongle acted as NMT master. This setup allowed the system to update motor targets and read sensors at 100 Hz simultaneously for all motors.

To command the exoskeleton efficiently and continuously, a custom software interface was created to bridge the CAN-bus USB interface to the robot operating system (ROS), which was used as the main ecosystem for control. A forward kinematic model of the exoskeleton was defined and the MoveIt package [58], which relies on the Orocos Kinematics and Dynamics Library [59], was used to manage inverse kinematics and trajectory planning, thus enabling end-effector control. Live end-effector control of the exoskeleton was facilitated via the jog_control package [60]. See Figure 6 for a system overview.

### 3.2. Intelligent Control

**Figure 7:**
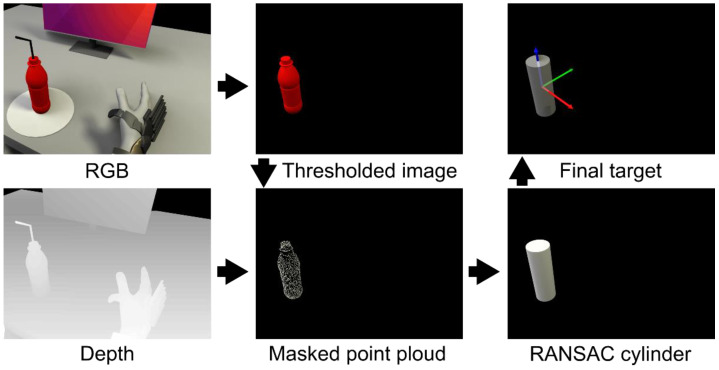
Image processing pipeline. A threshold is applied to the RGB image (**top left**), which results in a mask of all the red objects in the image (in this case the bottle, **top middle**). This mask is used to isolate the same objects in the point cloud (**bottom middle**), which is obtained from the depth image (**bottom left**). A random sample consensus method is performed to find the best fitting cylinder shape on the extracted point cloud object(s) (**bottom right**). The center of the found cylinder(s) is determined to be the target position (**top right**).

For the intelligent control to work, the essential task was to detect and determine the position and orientation of an object of interest in relation to the exoskeleton. To find the objects of interest, a computer vision algorithm was used to process the combined color and depth camera feed. The algorithm started by performing an initial thresholding operation in the HSV color space to extract red hues in the image, as we deliberately choose red objects as the objects of interest. By having the depth and color information aligned, the resulting mask of the red objects obtained from the thresholding operation could be applied to the depth map, and thus the depth maps of red objects were isolated. Subsequently, the masked depth map was converted into point clouds, thereby forming point clouds for each of the red objects in the original image. Each of these point clouds were evaluated using a random sample consensus (RANSAC) approach for their resemblance to a cylinder shape. The computer vision algorithm is illustrated in Figure 7.

Once the position and orientation of the object were known, the trajectory could be calculated and the motion to go from the current pose of the exoskeleton to a grasp pose around the object of interest could be performed. The trajectory from the current pose of the exoskeleton was planned continuously through the jog_package. The software packages used to extract the target object from the image and depth feed were OpenCV for thresholding and reading the incoming camera data and the Point Cloud Library [61] was used to perform RANSAC [62]. The computer vision algorithm and control method are described in greater detail in Bengtson et al. [63] (referred to as the “Fixed Semi-autonomous Control” scheme).

### 3.3. Tongue Control Interface Adaptations

Based on previous experiences and studies on optimizing the layout of the tongue interface [37,64,65], a weighted average of neighboring sensors approach was used to create a continuous sensing surface similar to a touchpad on a laptop. In a previous study from our group, this approach was found to achieve a throughput of 0.73 bits per second [37]. Additionally, a dwell time was used to prevent accidental activation during, e.g., speaking as found in a previous study [66].

Similar to Mohammadi et al. [65], a virtual joystick was implemented on the front surface of the mouthpiece to control the position of the hand in a 2D plane (forwards/backwards and left/right), see Figure 5c. Beneath the virtual joystick, a 1D virtual joystick controlled up/down, and the rear surface had buttons for opening and closing the hand, activating the autonomous control, and yet another 1D joystick to control the wrist rotation. This provided the user with direct, manual, and continuous control of the movements of the exoskeleton. The auto grasp button activated the intelligent control for approaching an object as long as the button was activated.

### 3.4. Test of the EXOTIC Exoskeleton

To test how the exoskeleton worked in activities of daily living, two studies were conducted. One five-day study was conducted with able-bodied individuals, and another three-day study was performed with users with tetraplegia.

#### 3.4.1. Participants

##### Able-Bodied Individuals

Ten able-bodied individuals (one female, mean age: 26.3 ± 4.8) were recruited for this study. The main exclusion criteria were severe right arm injuries, cognitive impairments, and drug addiction. Able-bodied participants were reimbursed for their time with DKK 100 per hour (equivalent to EUR 13.45 per hour) subject to income tax.

##### Individuals with Tetraplegia (Users)

Three individuals with tetraplegia (all male, mean age: 44.7 ± 19.1) were recruited for the study. Main inclusion criteria for individuals with tetraplegia were: (1) between 18 and 75 years of age; (2) reduced or absent motor function in the right arm (tetraplegia) caused by a spinal cord injury (ASIA impairment scale score of grade A to D) or ALS; (3) not able to repeatedly use the right hand and arm to grab a bottle with a straw (300 g) from a table and drink from it while in a seated position, and finally; (4) at least have some tongue functionality.

User 1 was able to flex and extend the elbow against gravity but had little to no function in wrist and fingers (ASIA: D, C4 injury), user 2 had a good arm function but little to no function of the fingers and wrist (ASIA: A, C5 injury). User 3 had no functional use of the upper limbs and depended on a ventilator (ASIA: C, C2 injury). Thus, this user had complete functional tetraplegia. INCSCI assessment results for each individual are available in Table 3.

#### 3.4.2. Experiment Description and Setup

##### Able-Bodied Individuals

The able-bodied individuals attended a five-day study comprising a series of training sessions and experiments with the EXOTIC exoskeleton system. The data shown in this paper were assessed at the end of the five-day study. The five-day study was split into two segments: three consecutive days followed by two consecutive follow-up days approximately one month later. Each session consisted of approximately two hours of focused use of the EXOTIC exoskeleton system. The initial three days tested the manual tongue control of the exoskeleton with two different tongue control methods [67], whereas the fourth and fifth sessions added semi-autonomy. The data presented in this article represent the performance of the able-bodied participants at the end of the final session (session 5).

The able-bodied participants were instructed to relax their arm and hand as much as possible during the experiments to simulate a paralyzed limb. To verify their cooperation with this request, eight surface electromyography (sEMG) electrodes (Myo Armband, Thalmic Labs Inc., Kitchener, ON, Canada, 2013–2018) were mounted on the arm on the transverse line between the medial acromion and the fossa cubit at 1/3 of the distance from the fossa cubit (approximately the peak of the biceps muscle). An sEMG recording of the max contraction of the participant was collected before mounting the exoskeleton. From this recording, a threshold was determined as 1/5 of the maximum contraction, which, if passed, would give the experimenter a warning during the tests such that the experimenter could remind the participant to relax the muscles.

##### Individuals with Tetraplegia (Users)

The experiment with the users comprised three sessions in total. The first session consisted of tongue controlling a simulation of the exoskeleton running on a computer. The extent of the spinal cord injuries for each user was determined through an ISNCSCI assessment performed by a trained medical doctor. In the second session, the users trained using the exoskeleton. Finally, semi-autonomous control was added to the experiment in the third session. The data presented here are from the last part of the final session. The first and second sessions consisted of approximately 1½ h and the last session approximately 2½ h of focused exoskeleton control. Following the tests, a short semi-structured interview was conducted with the users to obtain feedback from and opinions on their experience with the EXOTIC exoskeleton system. The interviews were recorded and later transcribed.

#### 3.4.3. Experiment Setup

The commercial version of the iTongue tongue control interface has active elements embedded in an acrylic palate brace with custom-fitted prongs that secure it to the teeth. However, in this study, a dental two-component A-silicone putty (Top Dent ImpressA Putty Soft, DAB Dental AB, SE) was used to create a temporary palate brace for the users to accommodate reuse of the system, see Figure 5b. Furthermore, a temporary activation unit was glued to the tongue of the participants as opposed to the medically inserted activation unit used for the iTongue commercial interface. The temporary activation unit was a 5 mm titanium sphere with a flattened top and bottom, see Figure 5b. The unit was glued near the tip of the tongue using a surgical skin adhesive (Histoacryl^®^ B. Braun Surgical S.A., Rubí (Barcelona), Spain).

For both users and able-bodied individuals, the custom silicone mouthpiece was molded during the first session. It was created by pressing the two-component silicone putty containing the tongue interface up against the palate of the individuals until the putty solidified (approximately 2 min). Before donning the exoskeleton, the exoskeleton links were adjusted to match the body size of the wearer. Likewise, an appropriate CarbonHand exoskeleton glove size was used for each individual. Additionally, to avoid errors related to slightly different hand mounting on the exoskeleton, the hand position was calibrated by moving it to a grasping pose on an already tracked object of interest. Knowing the position of the object, the position of the hand could be corrected in the kinematic chain such that when activating the autonomous function there would not be any offset issues. The activation unit was glued to the tongue as the last step before commencing the experiment.

**Figure 8:**
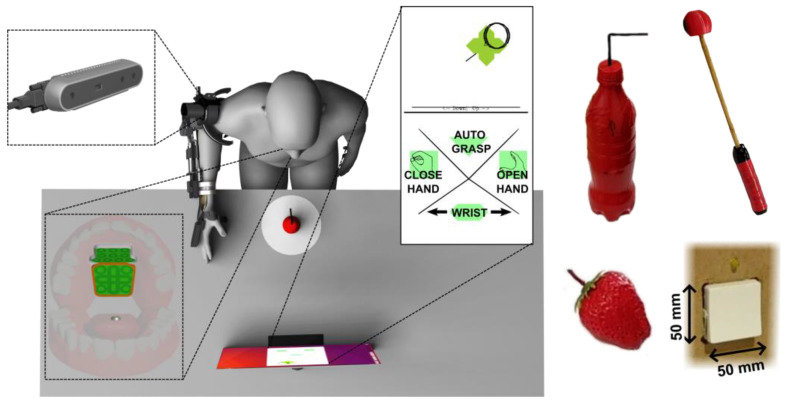
Experimental setup overview. The participant was positioned in front of a table with a bottle positioned 10 cm away from the table front. The iTongue system was mounted at the palate of the participant and the activation unit was glued to the tongue. A screen on the table showed dynamic visual feedback of the control layout and the position of the activation unit on the control layout. The objects used for ADL tasks are pictured on the right. From the top left: the bottle, the scratch stick, and the strawberry.

Each participant was seated in a chair, wheelchair, or powered wheelchair in front of a height-adjusted table in such a way that when the exoskeleton was mounted correctly, the wrist of the exoskeleton would be directly above the table edge, see Figure 8. A face shield was mounted to the head of the participant due to safety concerns.

#### 3.4.4. Experiment Tasks

The four ADL tasks used to evaluate the performance of the EXOTIC exoskeleton system for both able-bodied individuals and individuals with tetraplegia were: (1) the bottle task: grabbing a bottle with a straw from a table and moving it towards the face to make the straw touch the face shield (straw was 10 cm long above the lid, straight for able-bodied individuals and with a 90°, bend at 5 cm for individuals with tetraplegia to accommodate a more slanted sitting posture); (2) the strawberry task: grabbing a strawberry from a table and moving it to the face shield; (3) the scratch stick task: picking up a mock-up scratch stick from the table and moving it to make the end touch the side of the face or the face shield, and finally; (4) the switch task: depressing a standard Danish wall outlet switch (LK Fuga^®^, wall outlet switch 542D6001, 50 × 50 mm rocker switch surface) mounted to the right of the participant. As shown in Figure 8, the bottle was upright, whereas the strawberry and scratch stick was lying on the table. The end of the scratch stick was a ball made from soft material in order not to cause any harm in case of errors. The strawberry was an artificial plastic strawberry. The three grasp-and-transfer task objects were placed at a marked spot 10 cm from the table front centered in front of the user. A screen showing dynamic visual feedback from the tongue control interface was placed approximately 50 cm from the table front, see Figure 8. The switch was located 35 cm above the table, 10 cm from the table front, and 5 cm to the right of the exoskeleton shoulder.

Each task was performed three times and the averages of the three trials were used to obtain the results presented here. During the trials, the exoskeleton was configured to move at 4.5 cm/s and was configured to start at a predefined rest position, see Figure 8. If an object was dropped during a trial performed by able-bodied individuals, the trial was restarted. However, during trials performed by users, the objects were picked up by an experimenter and replaced in the hand while the user continued as if the object was still held in the hand. The reason for this discrepancy between the able-bodied individuals and users was more frequent drops and a higher risk of fatiguing the users in case of restarting the tasks too many times. The time for these tasks was recorded for the grasping part of the task as measured from the first command issued on the iTongue until the “close hand” command. The transferring to the face part of the task was measured from the “close hand” command until the task was completed. For the three grasp-and-transfer tasks, all participants were instructed to use semi-autonomous control.

## 4. Results

All experimental participants were able to control the EXOTIC exoskeleton system directly and continuously to perform the desired ADLs; even in the case of complete functional tetraplegia and the use of a ventilator (user 3).

**Figure 9:**
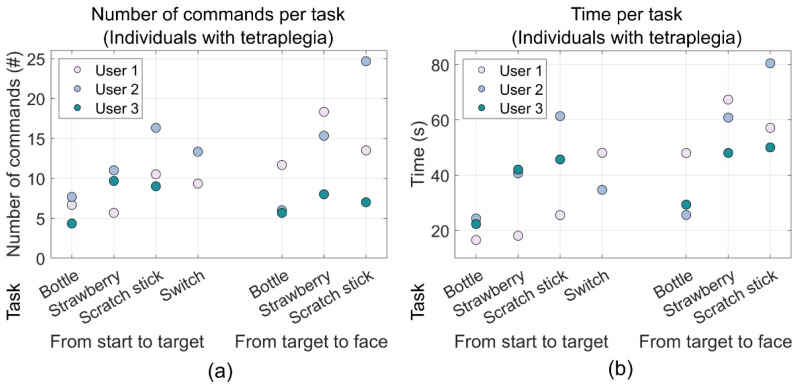
Scatter plots of the mean performance metrics for the individuals with tetraplegia (the users). The two groupings in each plot show the results from the reaching phase and the moving object phase, respectively, and each column corresponds to each task: bottle, strawberry, scratch stick, and switch, respectively. (**a**) Shows the number of commands used during each phase of each task while (**b**) shows the time it took to complete each phase of each task. The data for the switch task for one of the users (user 3) was unfortunately lost.

**Figure 10:**
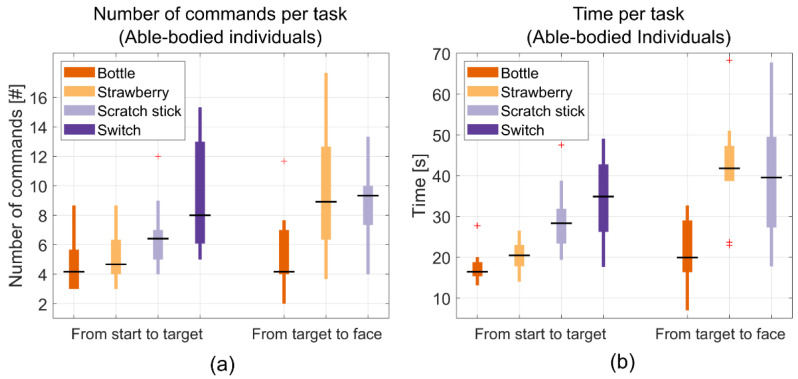
Boxplots of the mean performance metrics for able-bodied individuals. The two groupings in each plot show the results the reaching phase and the moving object phase, respectively, and each column corresponds to each task: bottle, strawberry, scratch stick, and switch, respectively. (**a**) Shows the number of commands used during each phase of the task while (**b**) shows the time it took to complete each phase of each task.

The able-bodied individuals were able to grab the bottle and move it to the face shield in 38.7 ± 6.1 s on average. The three users managed to perform the same task in 55.4 ± 8.0 s on average. When performing the increasingly difficult strawberry task requiring the wrist to be pronated while grabbing the strawberry and requiring the wrist to be supinated when reaching the face shield, the able-bodied individuals managed to perform the entire task in 62.7 ± 8.54 s on average. The same task was completed in 92.3 ± 11.6 s on average by the three users. The able-bodied individuals were able to perform the scratch stick task in 70.3 ± 12.0 s on average. The three users performed the same task in 106.7 ± 16.9 s on average. Finally, the switch task took 34.30 ± 10.78 s on average for the able-bodied individuals, while it took individuals with tetraplegia 41.39 ± 9.47 s on average. These results, along with the number of issued commands, are shown in Table 4 and as scatter and box plots in Figure 9 and Figure 10.

**Figure 11:**
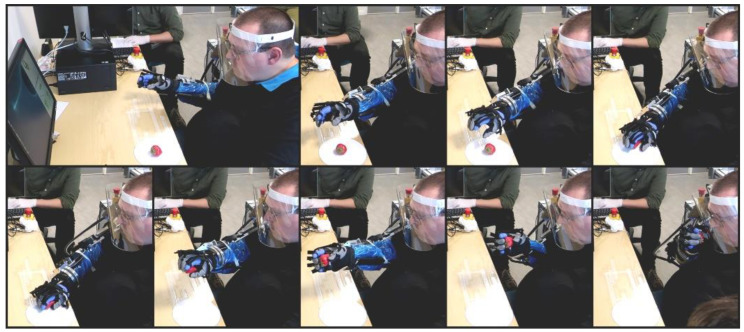
Image series of the strawberry task being performed by a user. The images show the initial approach, turn of the wrist, grasping and transfer to the mouth. Note: the blue plastic sleeve on the arm and the purple glove underneath the exoskeleton glove are only present due to the COVID-19 outbreak happening during the experiments.

During the four ADL tasks for the users, the soft exoskeleton glove could sometimes not apply sufficient force due to the added elastic bands which led to dropped objects. In these cases, the users were asked to continue as if the object was still in their hands. The objects were then placed back in the hand by an experimenter while continuing the motion of the exoskeleton, and if needed the object was supported by the experimenter until the task was completed. This was only observed during the user tests and not during the tests with the able-bodied individuals. As the exoskeleton glove was not the focus of this work, this was not considered for performance metrics. There were no occurrences of AD during the experiments. For an image series showing the strawberry task with a user, see Figure 11. For video sequences of the ADL tasks, see Video S1.

### Interviews

After ending the experiments, a semi-structured individual interview was conducted with each of the three users with tetraplegia. When questioned about how they experienced the functioning of the exoskeleton, the users indicated that the functioning was good. However, one of the users, user 1, who had some remaining arm function noted, “There is one function that I think I would like to have eventually. Currently, it can turn the wrist, but it cannot tip the wrist [abduction/adduction]. I think that will be missing if it is not there in the long run.” User 2 answered, “I think there is so much potential in this project. The freedom it would be to be able to pick up a bottle, drink from it yourself, and decide yourself. It would mean a massive difference. Function-wise, I think it is good, about where it should be.” User 3, who had the highest cervical damage and no arm function, agreed, “It has been great, great to be able to move the arm again—it was delightful.”.

When asked whether they could see themselves using it in their everyday life, the consensus was that the appearance should be more attractive, but they could all imagine using it. User 1 noted, “With respect to functioning and sound, I wouldn’t have second thoughts about using it, […] but I think it is unattractive.” When asked about the appearance, user 3 noted, “It looked big, but it would probably be smaller when attached to the arm [if it was attached directly to the powered wheelchair], so I think it is good at this point” and added that function and appearance are equally important. When questioned about the soft exoskeleton glove functionality, all users agreed that it needed substantial improvements as it simply could not supply enough grip force and spread between the fingers and the thumb.

The final questions revolved around the control method. The users, except user 3, agreed that the tongue control was tricky at first but noted that, “It will likely be easier with more time and training.” This fits well with prior experiences with learning curves [65] and the short time for training in this study.

## 5. Discussion

Through this work, we present the first shared, semi-autonomous, tongue-based upper limb exoskeleton system capable of fully assisting individuals with severe tetraplegia to perform multiple ADLs. In particular, this work shows the prospect for users with complete functional tetraplegia to be empowered through high DoF robotic devices by using a tongue control interface in combination with computer vision assistance as any possible motions of the compact arm and hand exoskeleton could be controlled directly and continuously by the users, thus facilitating a large variety of ADLs.

The EXOTIC exoskeleton was created to solve several challenges regarding full upper limb assistance, compact and mobile design, calibration and mounting time, ergonomic mounting to mitigate the risk of provoking AD, incorporation of user desires to perform certain ADLs, and finally to provide an effective, intelligent, and “invisible” control interface. These challenges were resolved by creating a novel five DoF exoskeleton with an ergonomic and “loose” mounting to the user and a workspace that included the space in which common ADLs could be performed. The control interface, the adapted iTongue, provided a concealed, flexible, and effective control of the exoskeleton shared with optional semi-autonomous functionality to assist in grasping. Despite using a screen for visual feedback in this study, the tongue interface can be used without it in trained users who memorize the control layout, akin to touch-typing on regular computer keyboards. The results from the tests performed in this study showed that it was possible to grab a bottle and move it to the face in less than one minute on average for both groups, whereas other more complicated tasks could be performed within two minutes in most cases. The users, who were interviewed after the experiment, endorsed the exoskeleton and attested the use of exoskeletons as assistive devices for individuals with tetraplegia.

During the user tests, it became apparent that the simple implementation of the soft exoskeleton glove chosen for this experiment exhibited some shortcomings that were not observed during tests on able-bodied individuals. The explanation for this discrepancy is most likely that the able-bodied individuals have flexible finger joints compared with the users, who exhibited contractures and spasticity in the finger joints. Thus, more advanced [22,28] or mechanically rigid solutions for grasping are recommended in future studies.

Further, the temporary silicone fitting used for the tongue interface in this study has a significantly larger size than the standard interface, which may have reduced the mobility of the tongue and may have affected the control efficiency and learnability as compared with the standard iTongue interface. The commercial iTongue unit would be a drop-in replacement for the modified device used in this study as they are technically identical, except for the software defining the layout of the commands (Figure 5c). The commercial version would be a safer, more comfortable, and likely an even easier device to use.

When comparing the able-bodied individuals with the actual users, a discrepancy between the two groups is clear. This discrepancy is probably due to the differences in demographics, prior training with the iTongue control interface, and the difficulties with the soft exoskeleton glove. Finally, the able-bodied individuals had a longer training period with the system and the age of the two groups was considerably different. These factors may well have caused the discrepancy. The differences observed would most likely diminish with more training, matched groups, and a better hand opening and closing mechanism. Further, a larger sample group of individuals with tetraplegia would better represent the actual performance of this group, though the number of participants is comparable to similar studies on individuals with complicated medical conditions [15,22,23]. An increase in the number of participants is often accompanied by a shorter experiment [22].

Nann et al. [22] demonstrated the use of state-based BMI control of a four DoF upper limb exoskeleton [23] with a five DoF wrist and hand exoskeleton. Their results showed that individuals with hemiparesis from stroke were able to execute a drinking task using their system. However, the users in that study still had arm functionality and the exoskeleton used was of a considerable size compared with the exoskeleton presented here. As indicated in the user interviews in our study, both functionality and appearance should be of high priority. BMIs have the advantage that they can be used by individuals without tongue function, but while the deployed state-based control for the BMI [23] may ensure better compliance in performing certain tasks, it reduces the flexibility considerably compared to the direct continuous tongue-based control employed in this work. All the users with tetraplegia participating in this study could imagine using the presented EXOTIC exoskeleton system in their everyday life.

## 6. Conclusions

Until recently, options for individuals with complete functional tetraplegia to control all motions of high DoF arm/hand exoskeletons continuously and directly have been practically non-existing. The results and the user feedback on the presented combination of an adapted available tongue control system, optional shared autonomous function, and a full compact and mobile arm/hand exoskeleton with a user-driven design indicate that this may be a viable solution to regain some independence and significantly increase the quality of life, even for individuals with complete functional tetraplegia.

## Figures and Tables

**Table 2:** Denavit–Hartenberg parameters of the EXOTIC exoskeleton. Parameters L_u_ and L_f_ correspond to the lengths of the upper arm and forearm, respectively, which are both adjustable.

Link	a_i_	α_i_	d_i_	θ_i_
1	0	π/2	0	π/2 − θ_1_
2	0	π/2	L_u_	π + θ_2_
3	0	−π/2	0	θ_3_
4	0	0	L_f_	θ_4_

**Table 3:** ISNCSCI assessment of users. Summary ratings of the ISNCSCI assessments for each user who participated in the study. * Indicates that the user used a ventilator.

	Age (Years since Injury)	Neurological Levels				Neurological Level of Injury	Complete/ Incomplete	ASIA Impairment Scale	Zone of Partial Preservation			
		Sensory		Motor					Sensory		Motor	
User		L	R	L	R				L	R	L	R
1	59 (0.6)	C4	C4	C5	C4	C4	I	D	NA	NA	NA	NA
2	52 (32)	C5	C5	C6	C7	C5	C	A	T11	L3	S1	S1
3 *	23 (0.7)	C2	C3	C2	C3	C2	I	C	NA	NA	L3	L3

**Table 4:** Metrics for the tests with able-bodied individuals and users. Mean and standard deviations of each metric of the described tests for both individuals with tetraplegia and able-bodied individuals.

	Able-Bodied Individuals				Individuals with Tetraplegia			
Measure	Bottle	Scratch Stick	Strawberry	Switch	Bottle	Scratch Stick	Strawberry	Switch
Time to object [s]	17.66 ± 4.13	29.66 ± 8.25	20.69 ± 4.09	34.30 ± 10.78	21.08 ± 4.05	44.17 ± 17.94	33.58 ± 13.44	41.39 ± 9.47
Time to reach mouth/ face shield [s]	21.02 ± 8.04	40.62 ± 15.76	42.05 ± 12.99		34.30 ± 12.00	62.50 ± 15.91	58.69 ± 9.82	
Number of commands until object [#]	4.60 ± 1.78	6.62 ± 2.41	5.10 ± 1.86	9.31 ± 3.95	6.22 ± 1.71	11.94 ± 3.87	8.78 ± 2.78	11.33 ± 2.83
Number of commands to face [#]	5.37 ± 2.82	9.10 ± 2.82	9.35 ± 4.33		7.78 ± 3.37	15.06 ± 8.94	13.89 ± 5.32	

## Data Availability

The data presented in this study are not publicly available due to privacy concerns.

## Supplementary Materials

The following supporting information can be downloaded at: https://www.mdpi.com/article/10.3390/s22186919/s1, Video S1: Demonstration of the experiment tasks using the EXOTIC exoskeleton.
